# OPHTHALMIA NEONATORUM in Italy: it is time for change

**DOI:** 10.1186/s13052-021-01186-2

**Published:** 2021-12-18

**Authors:** Cinzia Auriti, Vito Mondì, Salvatore Aversa, Daniele Merazzi, Simona Lozzi, Sergio Petroni, Antonio Baldascino, Luca Massenzi, Roberto Bellù, Stefano Martinelli, Fabio Mosca

**Affiliations:** 1 Neonatal Intensive Care Unit, Medical and Surgical Department of Fetus, Newborn and Infant – “Bambino Gesù” Children’s Hospital IRCCS, Rome, Italy; 2 Neonatology and Neonatal Intensive Care Unit, Policlinico Casilino Hospital, Rome, Italy; 3 Neonatal Intensive Care Unit, Children’s Hospital, ASST Spedali Civili, Brescia, Italy; 4 Division of Neonatology, ‘Valduce’ Hospital, Como, Italy; 5 Ophthalmology Department, Bambino Gesù IRCCS Children’s Hospital, Rome, Italy; 6 Ophtalmology Department, Fondazione Policlinico Universitario A. Gemelli, Catholic University of the Sacred Heart, Rome, Italy; 7 Department of Pediatrics and Neonatology, ‘S. Giovanni Calibita’ Fatebenefratelli Hospital, Rome, Italy; 8 Neonatal Intensive Care Unit, ASST, Lecco, Italy; 9 Azienda Socio Sanitaria Territoriale Grande Ospedale Metropolitano Niguarda, Milan, Italy; 10 Neonatal Intensive Care Unit, Fondazione IRCCS Ca’ Granda Ospedale Maggiore Policlinico, Milan, Italy; 11 Department of Clinical Sciences and Community Health, University of Milan, Milan, Italy

**Keywords:** Ophthalmia neonatorum, Prophylaxis, *Neisseria Gonorrhoeae*, Chlamidya trachomatis

## Abstract

Ophthalmia neonatorum (ON) refers to any conjunctivitis occurring in the first 28 days of life. In the past *Neisseria gonorrhoeae* was the most common cause of ON. It decreased with the introduction of prophylaxis at birth with the instillation of silver nitrate 2% (the Credè’s method of prophylaxis). Today, the term ON is used to define any other bacterial infection, in particular due to *Chlamydia Trachomatis*. Currently, the WHO reccomends topical ocular prophylaxis for prevention of gonococcal and chlamydial conjunctivitis for all neonates. On the contrary, several European countries no longer require universal prophylaxis, opting for screening and treatment of pregnant women at high risk of infection. And what about Italy? Have a look on Italian history of prophylaxis, starting by the first decree issued in 1940, signed by Benito Mussolini. In the following decades the law has undergone many changes. At the moment, legislation is unclear, therefore careful consideration is required in order to draft the correct appoach.

## Introduction

Ophthalmia neonatorum (ON) is a severe bacterial ocular infection occurring within the first four weeks of life from1 to 12% of neonates [1]. Traditionally the term mainly defined conjunctivitis from *Neisseria gonorrhoeae*. Today, it is used to describe any other form of neonatal ocular infection, in particular conjunctivitis from *Chlamydia trachomatis*. In the United States, *Neisseria gonorrhoeae* conjunctivitis has an incidence of 0.3 per 1000 live births, while *Chlamydia trachomatis* represents 8.2 per 1000 cases [2, 3]. The incidence of infection from both these pathogens has declined over recent decades, due to decreased prevalence in the population and to the introduction of prenatal screening. They are much more common in less developed countries. *Staphylococcus aureus*, *Streptococcus pneumoniae, Pseudomonas* and *Haemophilus* species and other Gram-negative bacteria, make up most of the remaining 30–50% of ON cases. Viral infections are less common and can be caused by herpes simplex virus, adenovirus or enterovirus.

The main risk factor for ON from *Neisseria gonorrheae* or *Chlamydia trachomatis* is the presence of a sexually transmitted infections (STI) in the mother. Before the routine use of topical eye prophylaxis with silver nitrate, tetracycline, or erythromycin an infant born from a mother with *Neisseria* endocervical infection had approximately from 30 to 50% of chance of developing ocular disease. A premature infant or an infant born after a prolonged period after membrane rupture has an even greater risk of developing infection [4–7].

Usually ON is a mild illness, but conjunctivitis due to *Neisseria gonorrhoeae* is a severe bacterial ocular infection [1]. Without any therapy the infection may progress quickly to corneal ulceration, perforation of the globe and permanent visual impairment [8]. Infants at increased risk for gonococcal ophthalmia are those whose mothers are at risk for STI [9]. Infants born to women with untreated *Chlamydia trachomatis* infection at delivery have a 50% chance of contracting the conjunctivitis and from 10 to 20% chance of developing pneumonia [10].

## The ocular prophylaxis HYSTORY

Before 1880 ON, mainly caused by *Neisseria gonorrhoeae,* was the leading cause of permanent blindness in neonates [12, 13]*.*. Then Dr. Credè, an obstetrician from Leipzig, reported the use of silver nitrate for prophylaxis against neonatal conjunctivitis. With a glass rod, he instilled a single drop of 2% silver nitrate into each eye of the newborn immediately after birth [11], reducing the incidence of neonatal conjunctivitis to zero. The Credè’s method of prophylaxis soon spread to the rest of Europe and to America; however, the concentration of silver nitrate was reduced to 1%, due to the frequent onset of chemical conjunctivitis [14, 15].

In the late 1970s the United States recommended 1% silver nitrate solution as the only pharmacological agent useful for the prophylaxis of ON. Later, many Countries (e.g. Denmark, Sweden, and the United Kingdom) suspended the obligation to ocular prophylaxis. The reason was that no substance is 100% safe for this purpose. Furthermore, the incidence of this infection among neonates does not justify the use of an universal prophylaxis. In fact the risk of contracting a sight-threatening infection with *Neisseria gonorrhoeae* is extremely low [16]. Prophylaxis was only recommended in infants whose mothers had specific risk factors. However, in the last twenty years *Chlamydia trachomatis* has progressively become widespread in many Countries and Crede’s prophylaxis has become a controversial issue because of its ineffectiveness against this germ. In addition, silver nitrate frequently caused chemical conjunctivitis [16–18]. For all these reasons silver nitrate has been replaced with 0.5% erythromycin or 1% tetracycline ointment in many Countries, for the greater efficacy of these drugs on the *Chlamydia trachomatis.* Povidone iodine 2,5% or 1% and fusidic acid were also tested and recommended as alternative drugs [19].

## Current guidelines

Several European Countries no longer require universal ocular prophylaxis, opting for the screening and treatment of pregnant women at high risk of infection.

For example, the Canadian Pediatric Society (CPS) does not recommend  universal prophylaxis with Erythromycin, the only drug currently available in this State, since 2015. CPS recommends that pediatricians and physicians, caring for newborns, should ask for stopping the requirement for eye prophylaxis, in Jurisdictions where it is still mandatory. They should promote epidemiological investigations to assess ON rates, allowing more targeted and effective preventive strategies [20].

In Countries where ocular prophylaxis is still implemented the debate on the first choice drug to be used is ongoing. In the United States, only the 0,5% erythromycin ophthalmic ointment is available among drugs approved by the US Food and Drug Administration. Other medications, such as tetracycline ophthalmic ointment and silver nitrate, are no longer available and Gentamicin was also reported as responsible for chemical conjunctivitis [21, 22]. In Brazil, the use of povidone iodine 2,5%, erythromycin 0.5%, tetracycline 1%, or silver nitrate 1% as rescue is recommended [23].

To date, WHO STI guidelines recommends topical ocular prophylaxis for prevention of gonococcal and chlamydial conjunctivitis for all neonates. It suggests the application on both eyes of: tetracycline hydrochloride 1%, or erythromycin 0,5%, or povidone iodine 2,5%, or silver nitrate 1%, or chloramphenicol 1% immediately after birth [24]. Indeed, WHO STI guidelines are aimed at all Nations of the world, including those at a low level of development, where strategies of targeted prophylaxis on mothers or on real-risk newborns are not often feasible.

## Current clinical practice in Italy

In 1940, the Prime Minister’s Office, Benito Mussolini, in a law on the midwives’ and nurses’ duties recommended that the midwife bag had to contain: a colored glass bottle with 5 ml of Silver Nitrate 1% solution in distilled water, or a solution of Protargol 5%, or Argyrol 15%, a full glass rod, 10 cm long, to instilling the drops in both the eyes of all neonates at birth, to prevent the ophthalmia neonatorum. *Since then in Italy all neonates at birth undergo antibiotic prophylaxis as the law is considered still ongoing* [25]. In 1975, a new law established that “the midwife , as soon as the birth has been completed and after having given the first care to the newborn, need to perform ophthalmic prophylaxis, according to the instructions of the Ministry of Health” [26]. This decree was then further repealed by a further law in 1999 that defines activities and responsibilities of the health personnel [27]. In the last law no mention has been made on the mandatory nature of this procedure, which seems to have been lost in the labyrinths of legislative renewal. In the Physiological Pregnancy Guidelines [28], drown up by the Ministry of Health in 2011, ophthalmic prophylaxis is described as effective and safe, but no drug was named and there are no recommendations relating to the mandatory nature of the administration to neonates at birth. Consequently, we have to go back to the international CDCs and WHO guidelines [22, 24], to read a clear recommendation on the neonatal universal ocular prophylaxis and specific drugs to carry it out, with the aforementioned limit of the universal meaning (nations with a high level of development and not) of the recommendation. In conclusion we are administering ocular antibiotics to all neonates at birth, looking to an Italian legislative obligation, that in Italy no longer has value.

## Some preliminary data on OPHTHALMIA NEONATORUM in Italy

With those information in mind, we carried out a quick questionnaire survey in the Italian birth centers, to which 29 centers responded. In all of them the prophylaxis is administered to all neonates at birth, with different drugs, at different times after the birth, within six hours of life of the neonate. In a population of 184,368 neonates, born in the last three years, only one conjunctivitis due to *Chlamydia trachomatis* and none is due to *Neisseria gonorroheae* were reported. We did not ask for the number of infections in the first 6 weeks of life.

## So what?

In 1994, in a “question time” session at the Ministry of Health, it was asked if a mandatory measure is not considered obsolete, considering the rarity of this infection in Italy. No suspensive measures have been decided but it is also true that accurate epidemiological estimates on these ocular infections among neonates in Italy are not available. In a historical phase in which great attention must be paid to the reasoned use of antibiotic therapies and prophylaxis, it is urgent to ask whether the administration of universal antibiotic prophylaxis is justified or not in our country.

 The careful monitoring of all pregnant women would be more effective? It should be noted  that what we currently do in clinical practice does not follow international guidelines  as drugs recommended in the WHO guidelines are not available in Italy. Choosing to follow the 2015 CDC recommendation, erythromycin 0,5% is the only antibiotic ointment recommended for use in infants. They report that silver nitrate ophthalmic ointment and tetracycline are no longer manufactured in the United States, bacitracin is not effective and povidone iodine has not been studied adequately. Gentamicin ophthalmic ointment has been associated with severe ocular reactions in neonates and should not be used for ocular prophylaxis. If erythromycin ointment is not available, neonates at risk of exposure to *Neisseria gonorrhoeae* (born from a mother at risk for gonococcal infection or without antenatal care) can be treated with ceftriaxone 25–50 mg/Kg intravenously or intramuscular, without exceeding 125 mg in a single dose. These guidelines also recommend routine screening and treatment of pregnant women and their partners during the first trimester of gestation. During the third trimester follow up and re-testing of the women considered at risk are recommended (multiple partners, less than 24 years of age, disadvantaged social status and so on). Regarding the usable products, silver nitrate is able to prevent ophthalmia neonatorum from *Neisseria gonorrhoeae*, however its use has been progressively abandoned due to the high risk of chemical conjunctivitis deriving from administration, which is observed in about 50% of treated infants.

Concerning mothers, CDCs recommend screening of pregnant woman at risk for *Chlamydia trachomatis* or *Neisseria gonorrhoeae* infection [22]..

Subjects considered at risk are:

- Women aged < 25 years and those aged > 25 years who have new sex partners
- More than one sex partner
- Sex partner with concurrent partners
- Sex partner who has STI
- Living in area with high prevalence of STI.

In the last Italian guidelines [28], *Chlamydia trachomatis* screening should be offered to pregnant women with recognized risk factors at first prenatal sight and should be repeated in the third trimester if necessary. Routine screening for *Neisseria gonorrhoeae* is not recommended in pregnant women but should be offered to women at risk of infection.

## Conclusion

In conclusion, in Italy we should target the ON prophylaxis at those neonates at risk, by using the appropriate drugs. At the same time we must consider that the flow of migration could increase the spread of these infections even in industrialized countries. Therefore, the screening should be widespread and include immigrant population.

## Data Availability

All data analyzed and used for the drafting of this article are available from the Corresponding author upon reasonable request.

## Abbreviation

ON: Ophthalmia Neonatorum
STI: Sexually Transmitted Infections
CPS: Canadian Pediatric Society
